# A case of massive hemobilia during plastic stent exchange following distal pancreatectomy with celiac axis resection and carbon-ion radiotherapy

**DOI:** 10.1055/a-2761-0620

**Published:** 2026-01-20

**Authors:** Koichi Soga, Masaru Kuwada, Ryosaku Shirahashi, Ikuhiro Kobori, Masaya Tamano

**Affiliations:** 1 26263 Department of Gastroenterology, Dokkyo Medical University Saitama Medical Center, Koshigaya, Japan

Hemobilia is a rare but potentially fatal surgical complication. Device insertion into a biliary system, either percutaneously or transpapillary, can predispose to arterial wall injury and pseudoaneurysm formation, occasionally leading to life-threatening hemobilia 1 2 . Here, we present a case of massive hemobilia during plastic stent (PS) exchange in a 52-year-old pancreatic cancer patient who had undergone distal pancreatectomy with celiac axis resection (DP-CAR) and carbon-ion radiotherapy, followed by adjuvant chemotherapy, 1 year prior.

**Fig. 1:**
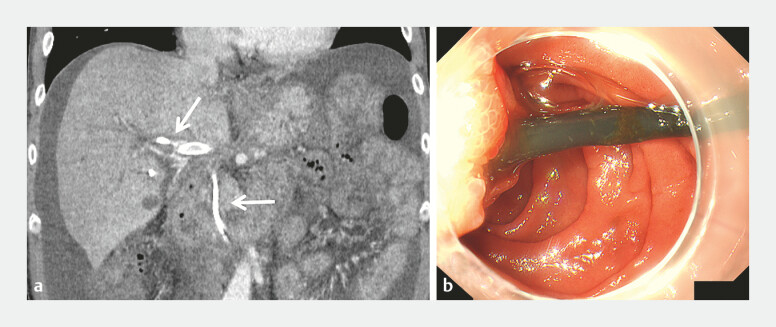
Imaging and endoscopic findings at admission. **a** The patient presented with melena, epigastric pain, and fever. Contrast-enhanced abdominal CT (arterial phase) showing the indwelling plastic stent (7 Fr × 10 cm; Piglet, Olympus, Japan) in the right intrahepatic bile duct (arrow). A previously inserted portal vein stent can be seen running parallel to the stent, and an artery arising from the right hepatic artery can be observed identified adjacent to the stent. **b** Upper gastrointestinal endoscopy performed to manage melena demonstrated blood efflux around the margin of the plastic stent, strongly suggesting hemobilia as the bleeding source. CT, computed tomography.

Three months prior to admission, the PS (7 Fr × 10 cm; Piglet, Olympus, Japan) was placed in the right B6 branch for biliary strictures. At admission, he presented with a 3-day history of high fever, melena, and chills. Laboratory findings revealed a hemoglobin decrease of 3 g/dL over several days, with mild jaundice and cholestatic enzyme elevation ( Fig. 1 ). Endoscopy excluded gastrointestinal bleeding, but blood adhering to PS suggested hemobilia.

**Fig. 2:**
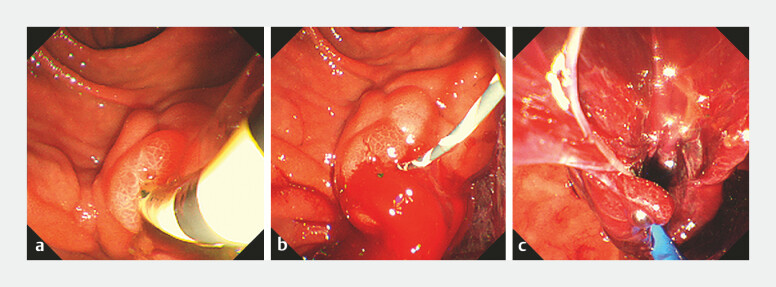
Endoscopic findings during stent removal and hemostatic management. **a** The previously placed plastic stent was carefully removed, as hemobilia was suspected as the bleeding source. **b** Immediately following removal, a massive pulsatile hemorrhage occurred from the bile duct orifice. **c** An endoscopic view during the insertion of the 7.5 Fr ENBD tube (Flexima, Boston Scientific, USA). The ENBD, which was slightly larger than the previous 7 Fr plastic stent, achieved effective tamponade and external drainage. No rebleeding or anemia progression was observed during the subsequent 2-week observation period, and the subsequent stent exchange was safely performed.

**Fig. 3:**
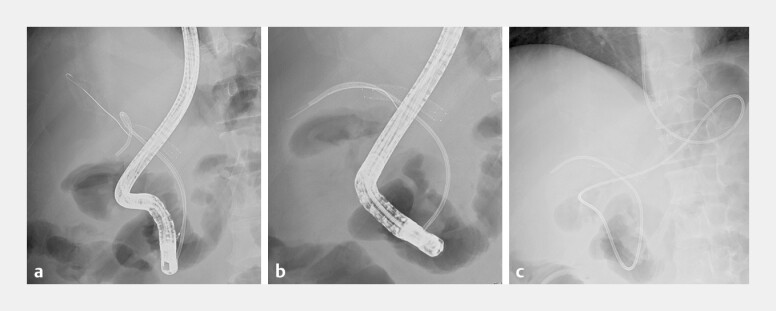
Fluoroscopic findings during endoscopic management of hemobilia. **a** Prior to plastic stent removal, a guidewire was carefully advanced alongside the indwelling stent to secure the biliary access, in anticipation of the risk of massive hemobilia. Because the patient had previously undergone DP-CAR, transarterial embolization would have been technically difficult if uncontrolled bleeding had occurred. **b** After pulsatile bleeding from the bile duct was observed, rapid intervention was performed. To ensure hemostasis, confirm bleeding control, and facilitate replacement with a larger caliber device, a 7.5 Fr ENBD tube (Flexima, Boston Scientific, USA) was inserted in place of the 7 Fr plastic stent. **c** A final fluoroscopic image taken after the successful ENBD placement, showing stable external drainage without any further bleeding.

During side-viewing duodenoscopy, blood efflux was observed around the PS. After guidewire placement, PS removal provoked sudden bleeding from the bile duct. A tapered 7.5 Fr endoscopic naso-biliary drainage tube (Flexima, Boston scientific, USA) was immediately inserted, providing drainage and tamponade. Hemostasis was achieved without transfusion or embolization, and no rebleeding occurred during 14 days of observation ( Fig. 2 , Fig. 3 and Video 1 ). Definitive stent replacement was performed safely. Because the patient had undergone DP-CAR, transarterial embolization would have been technically difficult, highlighting the importance of securing biliary access and an effective endoscopic strategy.

Massive hemobilia during plastic stent exchange following distal pancreatectomy in a patient treated with celiac axis resection and carbon-ion radiotherapy.Video 1

This case demonstrates that PS manipulation can precipitate massive hemobilia in post-DP-CAR patients with prior carbon-ion radiotherapy and chemotherapy. Vascular fragility and PS arterial compression likely predisposed to micro-pseudoaneurysm rupture. When visualization is poor, the temporary ENBD placement offers both decompression and hemostasis. Extreme caution is warranted when exchanging the PS in patients with altered vascular anatomy or prior oncologic therapy.

Endoscopy_UCTN_Code_CPL_1AK_2AC
